# Supplementary material for: Heavy Metals, Proximate Analysis and Brine Shrimp Lethality of Vernonia amygdalina and Ocimum gratissimum Growing in Crude Oil-Rich Delta State, Nigeria
Source: Foods. 2021 Nov 24;10(12):2913. doi: 10.3390/foods10122913 (PMC8701095; doi:10.3390/foods10122913)
Supplement: Supplementary file 1 [file foods-10-02913-s001.zip › foods-1445968-supplementary.pdf]

Appendix

Supplementary Table S1: Levels of the trace metals (µg/g, dry weight basis) in the analysed vegetable samples

**Vegetable samples**	**As**	**Cd**	**Cr**	**Pb**	**Hg**	**Tl**
VA-OAB	0.01± 0.00	0.00 ± 0.00	0.00 ± 0.00	0.02 ± 0.01	0.00 ± 0.00	0.01 ± 0.00
OG-OAB	0.01 ± 0.01	0.00 ± 0.00	0.00 ± 0.00	0.02 ± 0.01	0.00 ± 0.00	0.02 ± 0.01
VA-UAB	0.01 ± 0.01	0.00 ± 0.00	0.00 ± 0.00	0.01 ± 0.00	0.00 ± 0.00	0.03 ± 0.01
OG-UAB	0.01 ± 0.00	0.00 ± 0.00	0.00 ± 0.00	0.00 ± 0.00	0.00 ± 0.00	0.01 ± 0.00
VA-AAB	0.01 ± 0.00	0.00 ± 0.00	0.00 ± 0.00	0.00 ± 0.00	0.00 ± 0.00	0.00 ± 0.00
OG-AAB	0.01 ± 0.01	0.00 ± 0.00	0.00 ± 0.00	0.02 ± 0.01	0.00 ± 0.00	0.01 ± 0.00
VA-EGK	0.01 ± 0.01	0.00 ± 0.00	0.00 ± 0.00	0.02 ± 0.01	0.00 ± 0.00	0.02 ± 0.01
OG- EGK	0.01 ± 0.00	0.00 ± 0.00	0.00 ± 0.00	0.00 ± 0.00	0.00 ± 0.00	0.00 ± 0.00
VA-K	0.00 ± 0.00	0.00 ± 0.00	0.00 ± 0.00	0.03 ± 0.01	0.00 ± 0.00	0.01 ± 0.00
OG-K	0.01 ± 0.00	0.00 ± 0.00	0.00 ± 0.00	0.02 ± 0.01	0.00 ± 0.00	0.02 ± 0.01
VA-SK	0.01 ± 0.00	0.00 ± 0.00	0.00 ± 0.00	0.00 ± 0.00	0.00 ± 0.00	0.01 ± 0.00
OG-SK	0.01 ± 0.00	0.00 ± 0.00	0.00 ± 0.00	0.00 ± 0.00	0.00 ± 0.00	0.00 ± 0.00

VA- *Vernonia amygdalina*, OG – *Ocimum gratissimum*, OAB – Oria Abraka, UAB- Urhovie Abraka, AAB- Ajalom Abraka, EGK- Egbo Kokori, K- Kokori, SK- Samagidi Kokori. *Each value is a mean of triplicates ± SD. Means with no common letters within a column significantly differ ($p \leq 0.05$). n.d. = Not detected; $n = 3$.
